# Wound Healing as an Unconventional Marker for Epilepsy Control in a Patient Experiencing Homelessness: A Case Report

**DOI:** 10.7759/cureus.31052

**Published:** 2022-11-03

**Authors:** Jesse Obregon, Lauren DeLamielleure, Taha F Rasul, Brittany Blake, Armen Henderson

**Affiliations:** 1 Surgery, Ross University School of Medicine, Miramar, USA; 2 Biomedical Sciences, Florida Atlantic University Charles E. Schmidt College of Medicine, Boca Raton, USA; 3 Medical Education, University of Miami Miller School of Medicine, Miami, USA; 4 Internal Medicine, University of Miami Hospital, Miami, USA

**Keywords:** street medicine, social determinants of health (sdoh), ­wound healing, trauma and epilepsy, homelessness

## Abstract

Post-traumatic epilepsy is a complicated disease that remains challenging to treat even for patients who are able to access care regularly. People experiencing homelessness (PEH) represent a vulnerable demographic for neurologic disorders, especially due to gaps in care, limited resources, and low health literacy. This is a case of a 53-year-old male experiencing homelessness who was encountered by low-resource medical providers in an extra-clinical setting. His medical history was pertinent for a traumatic brain injury at a construction site a few years prior. He was diagnosed with post-traumatic epilepsy but was lost to follow-up due to being homeless and lacking health insurance. He also had a history of multiple hospitalizations secondary to seizures and did not consistently take his anti-epileptic medications. He was noted to have multiple facial wounds of unclear etiology. Upon further investigation, he complained of episodes of waking up on the sidewalk with facial injuries. The high-risk characteristics of his seizures prompted street medicine providers to quickly arrange an appointment with a primary care doctor. The process was further expedited by petitioning other local charitable organizations. He was later connected to a physician and re-prescribed levetiracetam 1000 mg twice daily for his post-traumatic epilepsy. After taking his medication regularly, his facial wounds were noted to have dramatic improvement. In this way, his medication adherence was measured as a function of his healing wounds since a lack of fresh wounds implied a lack of spontaneous seizures and subsequent reinjury. Low-resource medical providers caring for PEH in extra-clinical settings may necessitate using unconventional indicators to assess disease status.

## Introduction

Epilepsy is a complex neurological disorder characterized by the recurrence of unpredictable seizures. Nearly 70% of people who are diagnosed with the disorder are unable to pinpoint its cause, although a history of abnormal genes, stroke, and malformations of the brain are frequently linked to its onset [1]. One notable factor in epilepsy development is cerebral trauma, which can lead to post-traumatic epilepsy (PTE). In fact, traumatic brain injuries (TBI) are a leading cause of acquired epilepsy [2]. Each year, brain injuries severe enough to warrant hospitalization affect up to 500,000 persons in the United States [3].

People experiencing homelessness (PEH) are more prone to issues with medication adherence and sleep hygiene, and experience elevated levels of stress and alcohol use, all of which have been identified as initiating triggers for seizure development in epileptic individuals [4,5]. In comparison to the general housed population, the prevalence of epilepsy and its resulting morbidity is higher among PEH [6]. The probability of developing moderate to severe TBI is nearly 10 times higher in PEH than in the general population [7]. Seizures that are not well-controlled pose a serious risk of bodily harm and death. PEH are often too sick to independently coordinate their care and may rely on non-traditional healthcare providers such as street medicine clinicians for their medical needs [8].

Street medicine is defined as direct medical outreach for PEH living near sidewalks, encampments, and overpasses [8]. This is in contrast to a fixed physical location seen in most free clinic initiatives. Herein, we describe a case of PTE in an unsheltered patient and explore the diagnosis, treatments, potential improvements, and general approach to managing a high-risk patient in a resource-limited setting.

## Case presentation

A 53-year-old, unsheltered male approached a Miami-based street medicine team during regular outreach. His medical history mostly remains unclear with the exception of a work-related incident that resulted in a TBI in 2015. Subsequently, he complained of experiencing repetitive and recurrent seizures occurring on a weekly basis. The seizures became frequent enough that he lost his employment and health insurance and was forced to live on the streets. He stated that throughout the previous year, he required emergency department admission via ambulance at least 12 different times due to seizures. He was previously prescribed levetiracetam 1000 mg twice daily to manage his epileptic episodes but had been lost to follow-up due to homelessness. He also noted two major problems with his medication; firstly, he could not afford refills due to lack of insurance and had to ration accordingly; secondly, he tried to avoid adverse effects like severe drowsiness.

**Figure 1:**
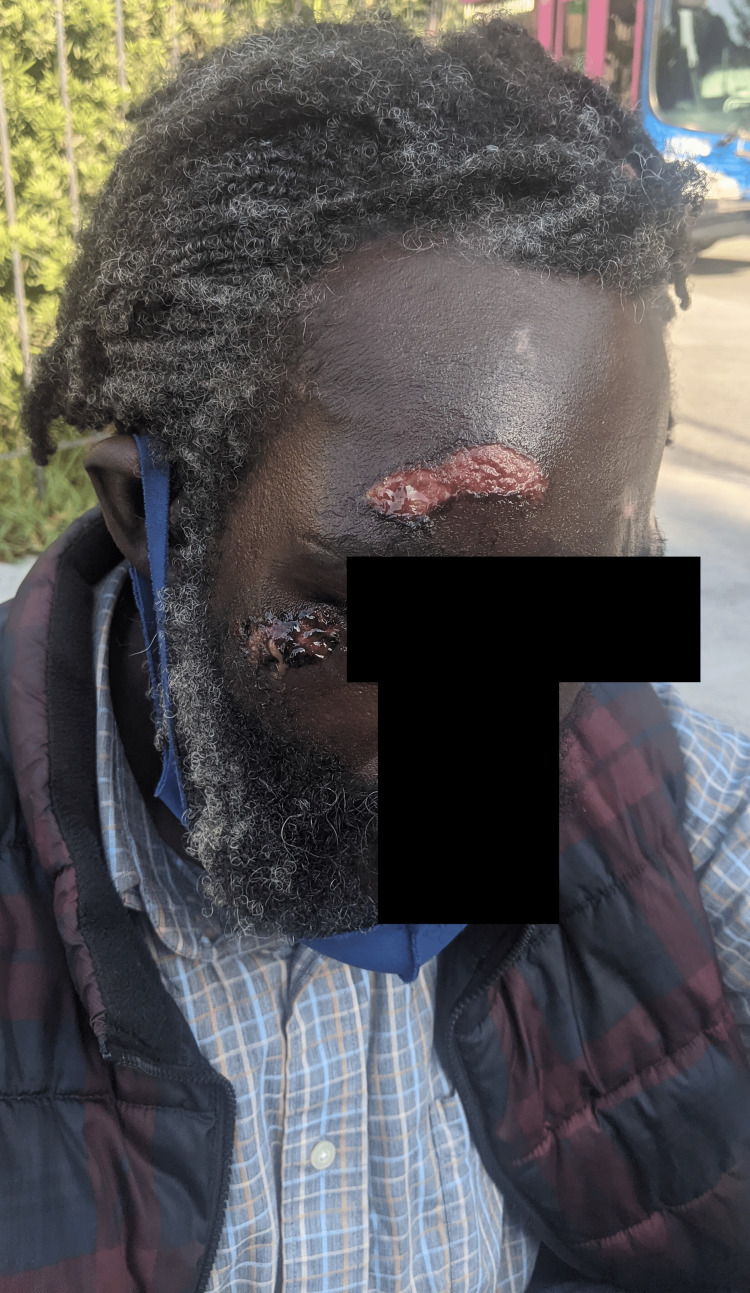
Initial patient evaluation demonstrating multiple face wounds The lesion on the right forehead was approximately 50 x 14 mm, whereas below the eye was roughly 29 x 15 mm. Photograph taken immediately after application of triple antibiotic ointment and petroleum jelly by street medicine providers.

On initial examination, his vital signs were within normal limits. His chief concern included the management of multiple facial lesions near his eye and around his face (Figure 1).

His wounds occurred during a recent unwitnessed seizure but he could not provide any further details. However, he explained this was not uncommon and reported several instances in which he woke up bewildered, lying on the concrete ground with fresh facial cuts and abrasions.

He was provided wound care in the form of petroleum jelly, triple antibiotic ointment, and bandages to cover the lesions. The triple antibiotic ointment was used for the wound with purulent discharge on the cheek, whereas petroleum jelly was applied to the wound on the forehead without overt signs of infection. Additionally, the street medicine team arranged for the patient to see a primary care physician (PCP) at a neighboring low-income clinic for additional testing and prescription refills. However, due to a lack of insurance and funds, further charity resources were used to expedite his care. The team also counseled the patient on adhering to his remaining medications to keep his epilepsy stable.

**Figure 2:**
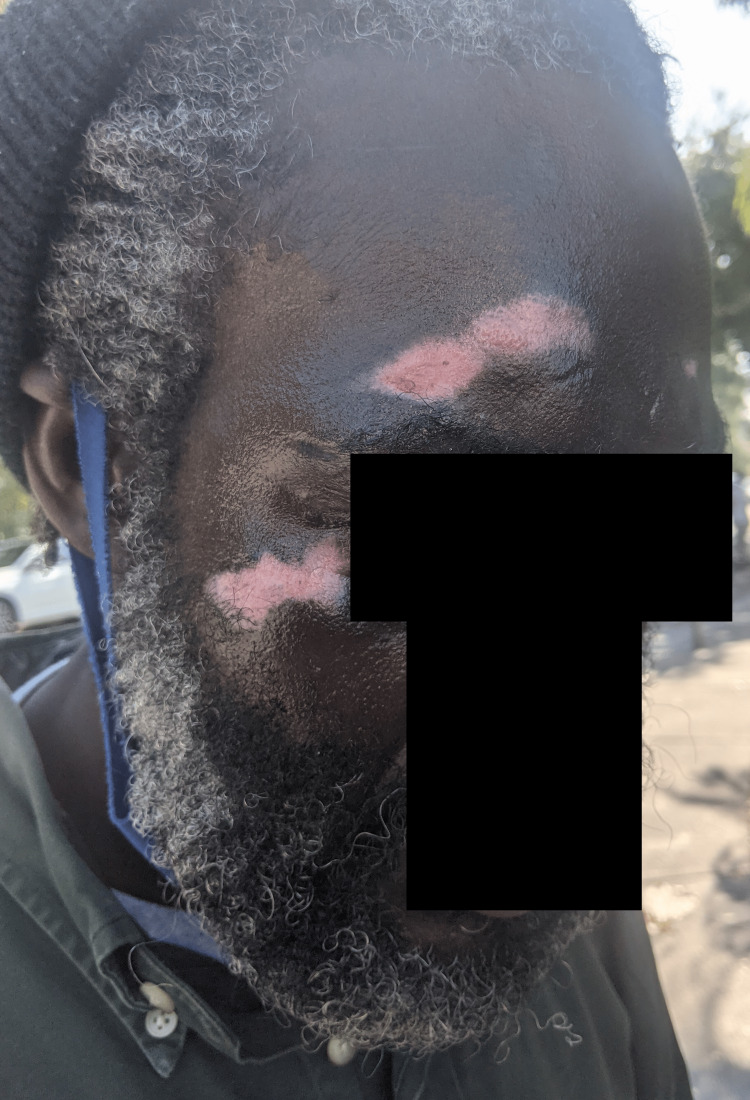
Two-week follow-up Both face wounds initially with granulation tissue healed toward re-epithelialization and no new lesions were noted. The lesion on the forehead was stable at 50 x 14 mm and the cheek lesion was 29 x 15 mm.

During subsequent outreach two weeks later, the street medicine team re-assessed the patient’s condition and facial lesions (Figure 2). There was a noticeable improvement in wound character, with appropriate healing of granulation tissue and re-epithelialization. He stated his levetiracetam had been successfully refilled, and he was now adhering to the treatment regimen, despite the medication making him feel fatigued. He was further counseled on the benefits of avoiding further trauma versus the risks of medication side effects and fatigue.

**Figure 3:**
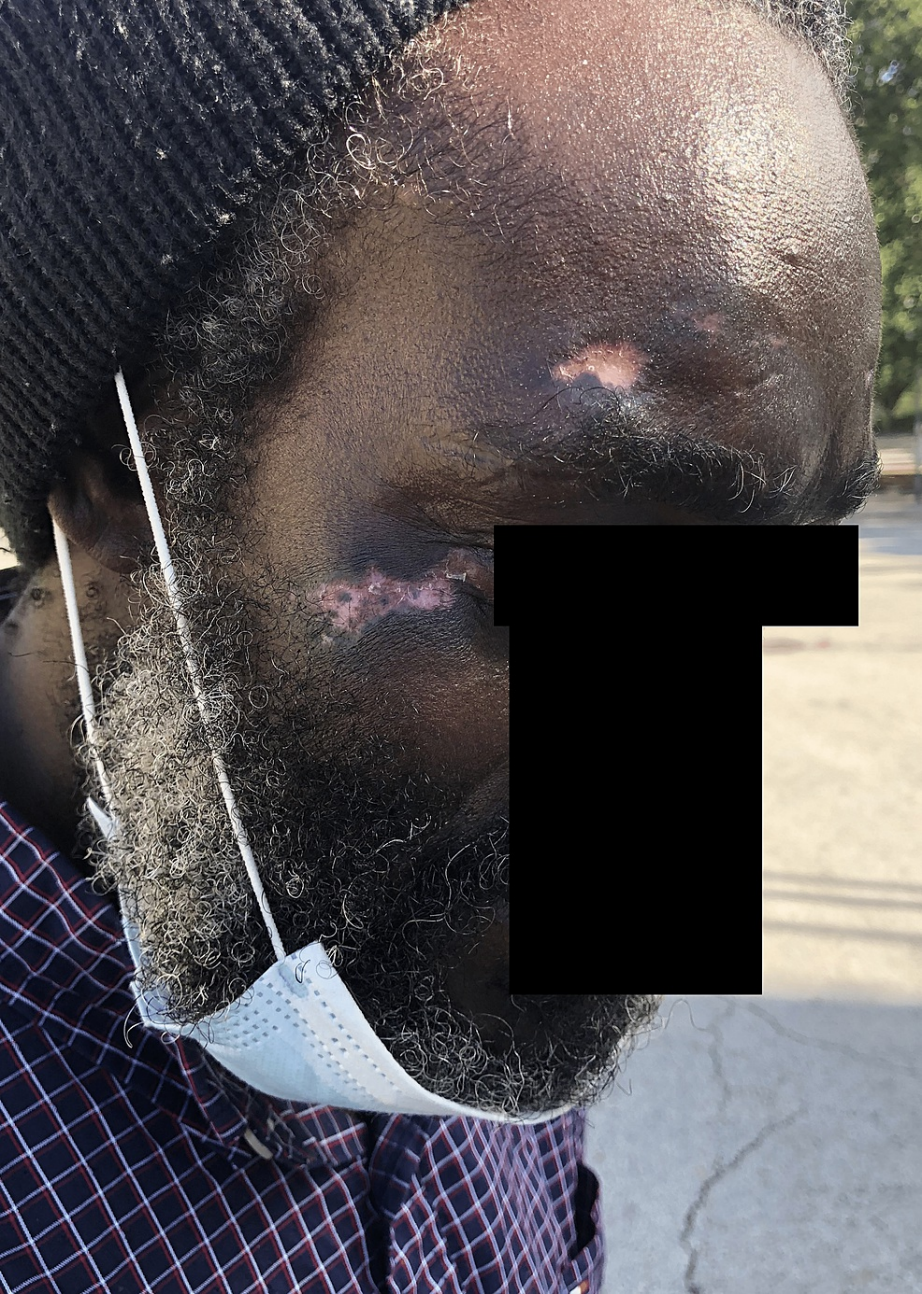
Four-week follow-up Repigmentation and shrinking of wound edges reduced the visible lesions on the forehead and cheek to roughly 20 x 11 mm and 29 x 13 mm, respectively.

Four weeks later, the patient was examined for follow-up. There was a noticeable improvement in his wounds with a shrinking of wound edges, repigmentation, and residual scar tissue (Figure 3). He also endorsed notably fewer epileptiform episodes, such as waking up on the sidewalk sporadically with no memory of prior events. The healing of the wounds acted as an indicator of improving seizure control and management. Additionally, there was no evidence of new bodily or facial injuries.

He continues to be followed up by the street medicine providers who further arrange primary care appointments and medication refills. Oftentimes, street medicine providers act as an intermediate point of care contact for unsheltered patients. This enables them to seek care outside of primary care appointments. Unfortunately, the follow-up has not been consistent and there are discrete times when our patient suffers from relapses, either due to his condition or medication non-adherence. His requirement for ambulance transportation and emergency department admission decreased to once in three months rather than at least once per month before. Overall, his condition has far improved compared to when he was first encountered.

## Discussion

This case highlights the multifaceted challenges of treating neurological disorders in PEH. Epilepsy is a relatively common, chronic neurological disorder with an estimated prevalence of five to eight cases per 1000 persons in industrialized countries, however, the epidemiology in homeless populations may be an area of further investigation. For PEH, this may present personal safety issues due to the inherent danger of falling on sidewalks, roads, and bridges. Additional risk factors for the development of PTE such as alcohol abuse are especially important to keep in mind since PEH often suffer from substance use disorders. A family history of epilepsy, an aging brain, and a genetic susceptibility to epilepsy exacerbated by TBI can all raise the chance of seizures [9]. Medical providers in low-resource settings could consider cost-effective methods of surveilling patients with signs of uncontrolled epilepsy, such as new onset wounds (which could be a sign of seizure-related falls) and reports of recent TBI.

Management of PTE is still a matter of active research and pharmacologic measures are often insufficient in complete control. Approximately one-third of epilepsy cases are refractory to medications and may require more invasive interventions like surgery, which is also associated with significant risks [10]. Additionally, it has been found that phenytoin is more cost-effective than levetiracetam for seizure prevention after TBI [11]. The likelihood of having additional seizures, brain damage, injuries, and early death increases with delayed diagnosis of seizure disorders and inadequate treatment [12]. For PEH lacking appropriate insurance or funds, expensive medications like levetiracetam can be out of reach. This was demonstrated in the case of our patient, who had to ration his medications after being lost to follow-up.

For patients who respond well to medication, effective management of epilepsy necessitates strict compliance with the treatment regimen. Non-adherence and abrupt discontinuation of medication are dangerous and can precipitate seizures, worsen patient outcomes, and antiepileptic drug side effects [13,14]. However, epilepsy management can be especially difficult for PEH because of the socioeconomic hurdles that disrupt consistent medication access and regimented use. Although levetiracetam is less of a cost-effective option in comparison to phenytoin, it is generally a safer drug in terms of side effects and medication interactions. Even though there is a lack of consensus, therapeutic levels are often between 20 and 40 mg/L [15]. Although routing monitoring is usually not indicated, it may be necessary to ensure a stable dose, detect potentially toxic levels, assess renal clearance, or even verify compliance. Symptoms of levetiracetam toxicity include dizziness and somnolence, with a small risk of respiratory depression [16]. Our patient's complaints of drowsiness were comparatively mild, and it was determined that he was likely in an appropriate therapeutic range. In the case of our patient with limited resources, directly measuring serum drug levels was not feasible, and wound improvement was used as a proxy for epilepsy improvement.

This unfortunate case demonstrates the shortcomings of the healthcare system in providing sustained primary care to vulnerable populations. From a practical perspective, the taxpayer burden of near-monthly ambulance transportation and emergency department admissions could have been greatly reduced if he were provided subsidized primary care appointments and medication refills. While delivery of care models continue to be developed in the United States, PEH may be left out of the conversation, worsening their already vulnerable health and forcing them to use non-traditional healthcare services like street medicine.

## Conclusions

Traumatic brain injury and resulting epilepsy were previously unmanaged in our unsheltered patient due to a lack of resources and consistent care. This case demonstrates a method for surveilling epileptic symptoms and medication compliance in the context of limited resources. Wounds sustained during seizure-related accidents can demonstrate disease severity and instability. The street medicine setting where PEH is treated can make it difficult to assess patients for neurological disorders without a proper work-up including imaging. This case demonstrates how in a limited-resource setting, monitoring facial wound healing processes served as an unconventional indicator of disease status. Resolving wounds, in addition to overall functional status indicated the patient’s seizure disorder stability.

Connecting this patient to a primary care physician by leveraging charitable resources helped in the improvement of his seizure disorder. Patients with limited health literacy or misconceptions about medication risks such as drowsiness may require further counseling and encouragement to stay adherent to their treatment plan. Street medicine teams can serve as a bridge for PEH from the street to the clinical setting. Providers treating PEH in resource-limited settings should determine whether wounds are associated with other underlying diseases.
